# ROS–SUMO Crosstalk in Oxidative Stress: Disease Mechanisms and Reproductive Health

**DOI:** 10.3390/antiox15040453

**Published:** 2026-04-04

**Authors:** Ann-Yae Na, Hyun-Shik Lee, Hong-Yeoul Ryu

**Affiliations:** 1 KNU G-LAMP Project Group, KNU Institute of Basic Sciences, School of Life Sciences, College of Natural Sciences, Kyungpook National University, Daegu 41566, Republic of Korea; 2 BK21 FOUR KNU Creative BioResearch Group, School of Life Sciences, College of Natural Sciences, Kyungpook National University, Daegu 41566, Republic of Korea

**Keywords:** human disease, oxidative stress, SUMOylation, reactive oxygen species (ROS), reproduction and fertility

## Abstract

Oxidative stress disrupts protein function through direct oxidation and triggers adaptive post-translational modifications. Among these, small ubiquitin-like modifier (SUMO)-ylation mediates fast and reversible remodeling of nuclear and cytoplasmic proteins. Redox regulation of the SUMO E1–E2 conjugation complex and specific SUMO proteases, such as SENP1 and SENP3, allows ROS to influence SUMO turnover and substrate selectivity. This defines SUMOylation as a versatile stress-response module under oxidative stress. In this review, we describe oxidative stress-induced remodeling of SUMO conjugation and deconjugation, with a focus on SUMO2/3 responses that transiently adjust transcription, DNA damage repair, and nuclear body dynamics. We discuss disease-relevant SUMO targets and pathological alterations in SUMO regulation across four major disease categories: neurodegenerative diseases, cardiovascular disease, cancer, and diabetes/metabolic diseases. In addition, we summarize emerging evidence connecting redox-sensitive SUMO remodeling to germ-cell function and reproductive health. Together, these perspectives highlight the dual role of SUMOylation as both a driver of stress adaptation and a tractable target for informing therapeutic strategies targeting the SUMO pathway.

## 1. Introduction

Oxidative stress can reprogram protein function on minute timescales, raising the question of which regulatory layers implement rapid and reversible proteome remodeling under stress [1,2]. Oxidative stress arises when reactive oxygen species (ROS) generation exceeds antioxidant capacity, perturbing redox homeostasis and damaging macromolecules and organelles; chronic imbalance causes degeneration, maladaptive remodeling, and loss of tissue function in multiple organs [3]. Dynamic post-translational modifications (PTMs) adjust protein activity, localization, aggregation propensity, and turnover in response to changing redox conditions [3]. Among these, small ubiquitin-like modifier (SUMO) has emerged as a key control module at the redox–proteome interface, and many stress paradigms are accompanied by an increase in SUMO-conjugated proteins, particularly in the nucleus, supporting a model in which SUMOylation acts as a versatile stress-response system [4,5,6].

SUMOylation is a reversible enzymatic cycle in which SUMO precursors are matured to expose a C-terminal di-glycine motif, activated by the SAE1/SAE2 E1 heterodimer, transferred to the sole E2 enzyme UBC9, and conjugated to lysine residues of substrate proteins, whereas SUMO-specific proteases (SENPs) reverse this process [7,8,9,10,11]. Mammalian cells express three principal SUMO paralogs, SUMO1 and the closely related SUMO2/3, which are not fully functionally equivalent [7,8,9]. In particular, SUMO2/3 more readily support chain formation and maintain a mobilizable free pool that can be rapidly engaged during stress, whereas SUMO1 is more often associated with mono-SUMOylation or chain-modifying functions [12,13,14]. This distinction is especially relevant in oxidative stress, where stress-induced SUMO remodeling often reflects SUMO2/3-dominant responses rather than a uniform increase across all SUMO paralogs [12,13,14].

Oxidative stress provides a powerful handle to dissect SUMO-mediated PTM crosstalk, since key components of the SUMO machinery are redox-sensitive [15]. Low H_2_O_2_ functions as a signaling metabolite that modulates pathways such as Nrf2/Keap1 and NF-κB, while higher or sustained ROS engages stress adaptation programs and can culminate in oxidative distress [16,17,18]. Mechanistically, ROS remodels SUMO turnover indirectly through kinase and ubiquitin–proteasome pathways and directly through the oxidation of catalytic cysteines in the SUMO E1/E2 enzymes and SUMO-specific proteases (SENPs) [16,17,18,19]. Conceptually, SUMOylation should therefore be viewed not only as a regulator of protein activity but also as a determinant of protein fate within the proteostasis network. Through its crosstalk with ubiquitination and SUMO-targeted ubiquitin ligases, SUMO can alter substrate stability by intersecting with ubiquitination, promote SUMO-targeted ubiquitin ligase-dependent turnover, or reshape broader quality control responses linked to protein aggregation, degradation, and autophagy-associated stress adaptation [10,20].

Consistent with these mechanisms, dysregulated ROS and altered SUMOylation are implicated in a broad spectrum of human diseases, including neurodegenerative [21] and cardiovascular disorders [22], cancer [23], diabetes and other metabolic diseases [24], and infertility [25]. Emerging evidence further indicates that altered SUMOylation contributes to disease progression across these settings by rewiring proteostasis, DNA damage responses, inflammatory signaling, and metabolic adaptation [21,22,23,24,26]. For example, SUMO2/3 responses in the nucleus coordinate transcriptional shutdown and DNA damage repair under ischemic or oxidative insults [27,28,29,30,31,32], whereas longer-term remodeling of SUMO pathways in the heart, tumors, metabolic tissues, and the reproductive system influences progression to heart failure, malignant transformation, diabetic complications, and subfertility [22,23,24,25].

In this review, we summarize evidence that SUMOylation functions as a reversible and redox-sensitive PTM that modulates cellular pathways central to human disease in oxidative environments [26]. We first outline how oxidative stress controls SUMO conjugation and deconjugation at the levels of enzymes, substrates, and nuclear structures, and we frame these interactions through a set of outcome-defining gates that connect ROS to kinase signaling, DNA damage responses, proteostasis, and hypoxia–mitochondrial adaptation. We then discuss oxidative stress-associated conditions spanning neurodegenerative, cardiovascular, metabolic, inflammatory, and malignant diseases, and we highlight emerging roles in reproductive health, where accumulating evidence suggests that ROS–SUMO crosstalk can shape sperm quality, meiotic chromosome behavior in oocytes, and the stress resilience of early embryos with potential consequences for fertility and reproductive aging. Finally, we consider how the dual nature of SUMOylation, as either a safeguard or a facilitator of pathology depending on context, creates opportunities and challenges for therapeutic targeting of ROS–SUMO signaling in human disease.

## 2. The SUMOylation Machinery

The SUMO family comprises several closely related peptides; SUMO1–3 are the principal paralogs, whereas SUMO4 and SUMO5 show more restricted or context-dependent expression [7,8,9]. SUMO resembles ubiquitin in overall architecture, but conjugation proceeds through a dedicated enzymatic cascade. SUMO is covalently attached to substrates through an isopeptide bond between the SUMO C-terminal glycine and a target lysine, frequently within the ΨKxE/D consensus motif (Ψ, bulky hydrophobic residue) [10,11].

**Figure 1:**
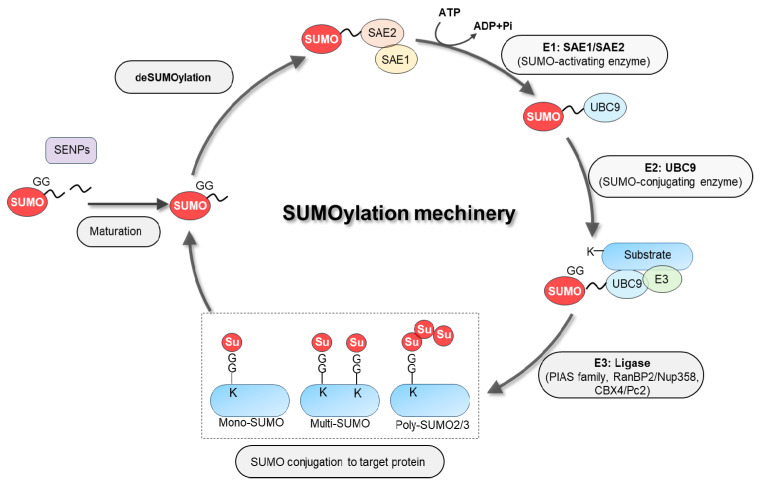
SUMOylation machinery. SUMO proteins (SUMO1–3) are synthesized as precursors that are processed by sentrin-specific proteases (SENPs) to expose the C-terminal di-glycine motif. The E1 heterodimer SAE1/SAE2 activates SUMO in an ATP-dependent reaction and transfers it to the conjugating enzyme UBC9, the sole SUMO E2. UBC9, assisted by E3 ligases such as PIAS family members, RanBP2/Nup358, and CBX4/Pc2, catalyzes SUMO attachment to lysine residues—often within a ΨKxE/D consensus motif—on a wide range of nuclear and cytoplasmic substrates. SUMOylation can occur as mono-SUMOylation or as SUMO2/3 chains, and mixed SUMO architectures intersect with ubiquitin signaling. SENPs remove SUMO and edit SUMO chains, thereby shaping SUMO turnover and paralog-specific signaling.

SUMOylation is driven by SUMO-specific E1, E2, and E3 enzymes and reversed by SENPs, allowing dynamic control of SUMO signaling (Figure 1) [10,11,33,34,35]. SUMO precursors are first processed to expose the C-terminal Gly–Gly motif, then activated by the E1 enzyme complex SAE1/SAE2 in an ATP-dependent reaction. Activated SUMO is transferred to the E2 enzyme UBC9 and, with the assistance of E3 ligases such as the PIAS family, RanBP2/Nup358, and CBX4/Pc2, is conjugated to specific lysine residues of substrates via an isopeptide bond. This results in mono-SUMOylation, multi-SUMOylation, or poly-SUMO2/3 chain formation in which SUMO itself becomes ubiquitin-like chain building blocks [10,12,13].

Mono-SUMOylation can generate regulatory surfaces that control genome stability, cell-cycle progression, and subcellular transport. SUMO2/3 also support chain formation (Lys11), and mixed SUMO architectures further diversify signaling outputs [12,13]. SUMO2 and SUMO3 are highly homologous, and the free SUMO2/3 enables rapid mobilization of conjugation during stress [14]. SUMOylation intersects with other PTMs, particularly ubiquitination, allowing cooperative or competitive control at shared lysine residues [10,20].

## 3. SUMOylation Responses to Cellular Oxidative Stress

Oxidative stress dynamically reconfigures SUMO signaling, coupling changes in SUMO conjugation to transcriptional control, DNA damage signaling, and nuclear proteostasis. In the nucleus, SUMO2/3-dependent responses to oxidative stress coordinate global changes in SUMO conjugation with transcriptional control, DNA damage signaling, and PML nuclear body-associated proteostasis. Quantitative proteomics and SUMO target profiling have shown that oxidative insults can markedly redistribute SUMO substrates, consistent with broad remodeling of the SUMOylated proteome under stress [14,36]. In oxygen–glucose deprivation (OGD), a cellular ischemia model, SUMO2/3 modification of transcription-associated proteins has been reported, and SUMO-dependent remodeling is most prominent during the stress phase and can partly reverse after restoration, highlighting the dynamic nature of this pathway in metabolic stress.

At the level of individual transcription factors, E2F transcription factor 1 (E2F1) is a clear example of stress-protective SUMOylation. Oxidative stress promotes the conjugation of SUMO2 to E2F1, primarily at Lys266, which dampens E2F1-dependent transcription and reinforces a G1/S arrest program. Disrupting E2F1 SUMOylation weakens arrest and increases sensitivity to oxidative stress. Together, these observations indicate that SUMO2/3-dependent modification of transcriptional regulators can transiently suppress transcription and proliferation, thereby limiting damage and allowing time for recovery [27].

**Figure 2:**
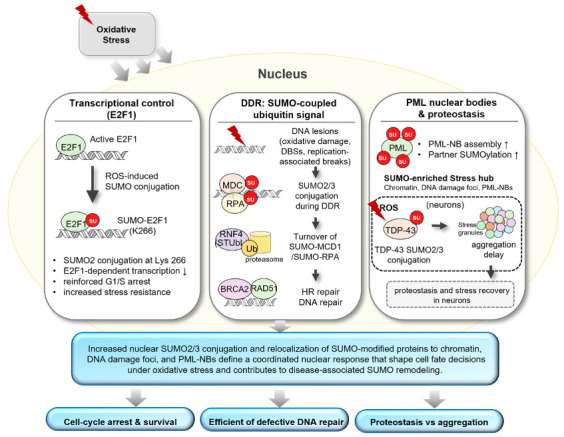
SUMOylation responses to oxidative stress. Oxidative stress activates nuclear SUMO2/3-dependent pathways, highlighting three representative modules: SUMOylation of E2F1 reinforcing G1/S arrest, SUMO–RNF4 coupled turnover of DDR factors enabling ordered homologous recombination, and SUMO-enriched PML nuclear bodies.

ROS generate oxidative DNA lesions that can be converted into double-strand breaks (DSBs) and replication-associated damage during DNA replication, thereby activating the DNA damage response (DDR) [28,29]. During DDR, the mediators of DNA damage checkpoint 1 (MDC1) and replication protein A (RPA) become SUMOylated, which recruits the SUMO-targeted ubiquitin E3 ligase RING finger protein 4 (RNF4) [30,37]. RNF4 then drives the proteasome-linked turnover of MDC1/RPA at damage sites, enabling their timely replacement by homologous recombination (HR) factors such as BRCA2 and RAD51 [37]. Consistently, the loss of RNF4 disrupts this turnover, prolongs MDC1 and phosphorylated histone H2AX (γH2AX) foci, and impairs HR, indicating that SUMO-coupled ubiquitin signaling is required for ordered repair progression under oxidative stress [30,31] (Figure 2).

PML nuclear bodies (PML-NBs) represent another major nuclear platform for stress-induced SUMO signaling. Oxidative stress drives PML-NB assembly and enhances partner SUMOylation within these bodies, consistent with a role in organizing stress-responsive nuclear quality control and regulatory factor processing. In neurons, oxidative stress can also alter the SUMO conjugation of RNA-binding proteins and synaptic regulators. For example, TAR DNA-binding protein 43 (TDP-43) undergoes stress-induced SUMO2/3 conjugation, linking SUMO remodeling to proteostasis and stress recovery pathways in the nervous system [38,39]. Collectively, increased SUMO2/3 conjugation and relocalization of SUMO-modified proteins to chromatin, DNA damage foci, and PML-NBs define a coordinated nuclear response that influences cell fate decisions under oxidative stress (Figure 2). Together, these findings help explain how prolonged oxidative stress can gradually remodel SUMO pathways across diverse diseases including neurodegenerative disease, cardiovascular disease, cancer, diabetes, and metabolic disorders.

## 4. SUMOylation and Human Disease

**Figure 3:**
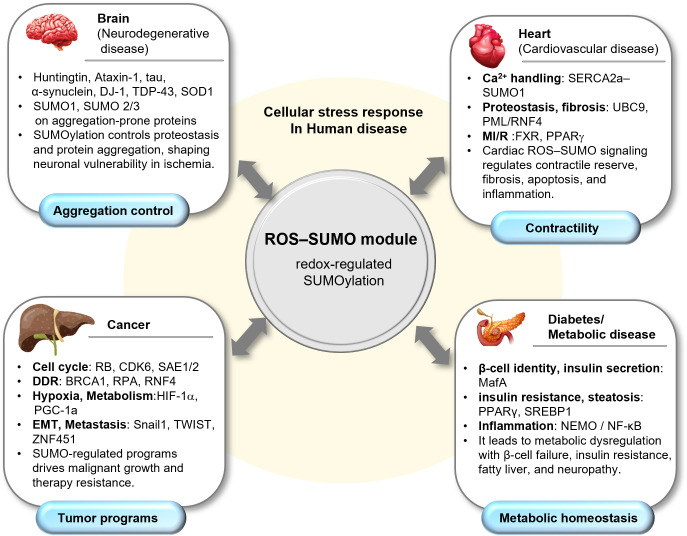
Organ-specific ROS–SUMO remodeling across human disease. Redox-regulated SUMOylation functions as a central module that is redeployed across organ systems, with disease-specific substrates and functions. In the brain, SUMO1 and SUMO2/3 modification of aggregation-prone proteins modulate proteostasis and neuronal vulnerability. In the heart, SUMO signaling influences Ca^2+^ handling (SERCA2a–SUMO1) and stress remodeling programs involving UBC9 and PML/RNF4, impacting contractility, fibrosis, apoptosis, and inflammation. In cancer, SUMO-dependent control of cell-cycle, DDR, hypoxia/metabolism, and EMT/metastasis support malignant progression and therapy resistance. In diabetes/metabolic disease, SUMO regulation of β-cell identity/insulin secretion (e.g., MafA), insulin resistance/steatosis (PPARγ, SREBP1), and inflammatory signaling (NEMO/NF-κB) contributes to metabolic dysregulation.

Several studies have linked SUMOylation to human disease mechanisms, particularly in disorders characterized by converging cellular stress, including oxidative stress. In the following sections, we discuss disease-relevant SUMO targets and pathological alterations in SUMO regulation that modulate stress tolerance and disease progression across four disease categories: neurodegenerative diseases, cardiovascular disease, cancer, and diabetes/metabolic disorders (Figure 3). Across these diseases, ROS–SUMO crosstalk converges on proteostasis, DNA damage responses, inflammatory signaling, and metabolic adaptation, whereas the principal SUMO-regulated substrates and downstream pathological consequences differ among tissues.

### 4.1. Neurodegenerative Diseases

Neurodegenerative diseases, including Alzheimer’s disease (AD), Parkinson’s disease (PD), Huntington’s disease (HD), spinocerebellar ataxia type 1 (SCA1), and amyotrophic lateral sclerosis (ALS), feature chronic proteostasis stress, mitochondrial dysfunction, and oxidative stress. These processes promote misfolding, aggregation, and selective neuronal vulnerability [21]. SUMOylation influences disease progression by altering the aggregation propensity, ubiquitin-coupled degradation, subcellular localization and stress-protective activity of disease-linked proteins. Many pathogenic proteins are direct SUMO substrates, and because SUMO and ubiquitin can target overlapping lysine residues, SUMOylation can redirect protein fate by shifting the balance between ubiquitylation-dependent turnover and persistence. In neurodegenerative disease, ROS–SUMO crosstalk is expressed primarily through proteostasis pathways, where SUMO-dependent changes in aggregation, ubiquitin-coupled turnover, stress-granule dynamics, and mitochondrial stress responses determine whether neurons preserve protein homeostasis or progress toward degeneration.

This section highlights representative SUMO targets organized by disease: huntingtin (HD), ataxin-1 (SCA1), tau (AD), α-synuclein and DJ-1 (PD), and TDP-43 and SOD1 (ALS), to illustrate connections between SUMOylation, protein aggregation, organelle dysfunction, and neuronal vulnerability.

#### 4.1.1. Ischemic Neuronal Injury

Transient cerebral ischemia and OGD model acute ischemic/energy-deprivation stress and reoxygenation-associated oxidative stress, with profound effects on neuronal viability. During ischemic episodes, global SUMO2/3 conjugation markedly increases, suggesting an adaptive stress response. Inhibition or depletion of SUMO2/3 worsens neuronal survival after OGD, supporting a broadly neuroprotective function for SUMOylation in a primary neuronal OGD model [32].

Mechanistically, SUMO cycling intersects mitochondrial dynamics during reperfusion stress. In dissociated primary cultured rat cortical neurons subjected to OGD/reoxygenation, SENP3-dependent deSUMOylation of DRP1 promotes mitochondrial fission and apoptosis following OGD/reoxygenation, whereas sustained SUMO conjugation limits mitochondrial fragmentation [40]. Together, these findings position the SUMO system as a critical modulator of ischemia-induced oxidative stress and mitochondrial dynamics, acting to preserve cellular homeostasis under energy-deprived conditions.

#### 4.1.2. Polyglutamine Diseases: Huntingtin and Ataxin-1

In the HD model, N-terminal huntingtin fragments (commonly HTTex1) are SUMOylated on N-terminal lysines that also serve as ubiquitin acceptors. A Drosophila study showed that the SUMOylation of HTTex1 exacerbates neurodegeneration, whereas ubiquitination at the same region reduces pathology, supporting functional competition between SUMO and ubiquitin in vivo [41]. A later study reported that SUMO2 and the SUMO E3 ligase PIAS1 (protein inhibitor of activated STAT 1) promote the accumulation of insoluble mutant huntingtin species, linking SUMO regulation to aggregate burden [42].

In SCA1, oxidative stress or c-Jun N-terminal kinase (JNK) pathway activation increases ataxin-1 SUMOylation and promotes aggregation, and JNK inhibition reduces oxidant-enhanced ataxin-1 SUMOylation and aggregation [43]. Thus, in polyglutamine disease models, SUMOylation promotes pathology primarily by favoring the persistence and aggregation of mutant proteins rather than their clearance.

#### 4.1.3. AD and PD: Tau, α-Synuclein, DJ-1

Tau provides a clear example of site-specific SUMOylation linked to turnover. Tau SUMOylation was demonstrated in transfected HEK293 cells expressing tau together with His-tagged SUMO isoforms, where tau was preferentially modified by SUMO1 at lysine 340 [44]. Proteasome inhibition increases tau ubiquitination while reducing tau SUMOylation, consistent with mutually exclusive occupancy that can influence degradation routes [44]. These findings support a model in which tau SUMOylation competes with ubiquitin-dependent processing and may thereby favor tau persistence under proteotoxic conditions.

α-synuclein shows a more variable direction across studies. One study reported that α-synuclein SUMOylation inhibits aggregation and toxicity in vitro and in vivo [45]. In contrast, another study reported that PIAS2-driven SUMOylation reduces α-synuclein ubiquitination and impairs degradation, thereby promoting accumulation and inclusion formation in cell-based systems [46]. These opposing results suggest that the consequence of SUMOylation depends on the extent to which SUMO-modified species are coupled to proteasomal or autophagic clearance pathways.

DJ-1 represents a distinct PD-linked mechanism because SUMOylation affects not aggregate formation but the integrity of a stress-protective protein. Cell-based studies showed that DJ-1 is SUMO1-conjugated at Lys130, and that proper SUMOylation is required for its growth-promoting, anti-apoptotic, and antioxidative functions. SUMO-defective DJ-1 mutants lose these protective activities, linking impaired SUMO control to reduced oxidative stress defense rather than to aggregate persistence per se [47,48].

#### 4.1.4. ALS: TDP-43 and SOD1

A recent neuronal study directly linked oxidative stress to SUMO2/3 conjugation of TDP-43. During oxidative stress, protein inhibitor of activated STAT 4 (PIAS4) mediates TDP-43 SUMO2/3 conjugation, promoting its enrichment in stress granules and delaying conversion into detergent-insoluble aggregates; the inhibition of SUMO conjugation or PIAS4 depletion accelerates aggregation in cellular neuronal models [39].

For superoxide dismutase 1 (SOD1), SUMO modification has been linked to the aggregation propensity of ALS-associated mutants. Studies report SUMO-dependent enhancement of mutant SOD1 aggregation, including SUMO3-related effects in motoneuronal cell systems [49,50]. Thus, for SOD1 mutants, the available evidence primarily supports a role for SUMOylation in aggregation kinetics rather than in protective stress adaptation.

### 4.2. Cardiovascular Disease

Oxidative stress is a central driver of cardiac remodeling, and SUMOylation regulates key proteins involved in calcium handling, protein quality control, fibrotic remodeling, and stress-responsive transcription. The dysregulation of this pathway is increasingly recognized as a contributor to heart failure, proteotoxic cardiomyopathy, cardiac fibrosis, and ischemia–reperfusion injury. The following sections highlight representative SUMO-dependent mechanisms involving SERCA2a, UBC9, the PML/RNF4 axis, and nuclear receptors in cardiac stress responses.

#### 4.2.1. SUMO1–SERCA2a in Heart Failure and Hypertrophy

SERCA2a, the sarcoplasmic reticulum Ca^2+^ ATPase in cardiomyocytes, is a well characterized SUMO target in heart failure. SUMO1 conjugation at two conserved lysine stabilizes SERCA2a and increases its ATPase activity, and these SUMO sites are hypomodified while SERCA2a levels are reduced in failing hearts [51]. In cardiomyocytes, the co-expression of SUMO1 extends the half-life of wild type SERCA2a, whereas the mutation of the two SUMO acceptor lysines shortens SERCA2a half-life and reduces its capacity to pump Ca^2+^ back into the sarcoplasmic reticulum [51]. Thus, the SUMOylation of SERCA2a increases protein stability and Ca^2+^ reuptake capacity, thereby helping preserve cardiomyocyte contractile function.

In transverse aortic constriction (TAC)-induced pressure overload in vivo, SUMO1 is a critical determinant of SERCA2a function and cardiac performance. SUMO1 knockdown accelerates the loss of SERCA2a activity, worsens contractile dysfunction, and increases markers of oxidative damage. By contrast, cardiotropic rAAV9-mediated SUMO1 delivery after the onset of heart failure increases myocardial SUMO1 content in a dose-dependent manner, enhances SERCA2a SUMOylation, reverses left ventricular dilation, improves ejection fraction, and prolongs survival, all without measurable changes in UBC9 or SENP1 levels [51]. Small molecule activators such as ginsenoside Rg3 likewise promote SERCA2a SUMOylation, improve Ca^2+^ cycling, and enhance ventricular performance in TAC-induced heart failure. Together, these findings support SUMO1-dependent SERCA2a modification as a core cardioprotective mechanism whose progressive loss likely contributes directly to reduced contractile reserve [51,52].

#### 4.2.2. UBC9-Dependent SUMOylation in Proteotoxic Cardiomyopathy

Cardiomyocytes are highly vulnerable to proteotoxic stress. They depend on long-lived contractile and mitochondrial proteins and have limited regenerative capacity. The SUMO E2 enzyme UBC9 controls global SUMO conjugation in the heart and has emerged as a key regulator of autophagy and protein quality control under stress.

Cardiac-specific UBC9 overexpression in mice increases overall SUMOylation in cardiomyocytes at baseline. Autophagic flux rises, as shown by increased LC3 II formation and autophagosome turnover in neonatal rat ventricular myocytes and adult hearts. These UBC9 transgenic mice maintain normal cardiac structure and function under resting conditions [53]. In a desmin related cardiomyopathy (DRM) model, cardiomyocyte restricted expression of mutant αB crystallin (CryABR120G) induces protein aggregation and impairs autophagy. The co-expression of UBC9 in CryABR120G hearts reduces the number and size of aggregates, attenuates myocardial fibrosis and hypertrophy, and significantly improves left ventricular function and survival compared with CryABR120G mice without UBC9 overexpression [53]. Mechanistic studies indicate that higher SUMOylation enhances clearance of misfolded proteins by both the autophagy–lysosome system and the ubiquitin–proteasome system. No overt toxicity of UBC9 overexpression has been reported in wild type hearts [54]. These findings suggest that UBC9-dependent SUMOylation defines the capacity of the myocardium to cope with aggregation-prone proteins. Pharmacologic strategies that increase cardiac SUMOylation may therefore help maintain proteostasis in inherited or acquired cardiomyopathies characterized by protein aggregation.

#### 4.2.3. PML/RNF4 Axis in Cardiac Fibrosis

Cardiac fibrosis is a common end point in pressure overload, myocardial infarction, and cardiotoxic drug exposure. It contributes to diastolic dysfunction and arrhythmia. The promyelocytic leukemia protein PML forms PML NBs and is heavily SUMOylated at multiple lysines. Poly-SUMOylated PML recruits SUMO interacting proteins and SUMO-targeted ubiquitin ligases such as RNF4 [55]. In this setting, sustained oxidative and neurohumoral stress converges on SUMO-dependent nuclear remodeling pathways that influence fibroblast activation and extracellular matrix deposition.

Recent studies have identified a UBC9/PML/RNF4-dependent regulation that controls cardiac fibroblast activation and extracellular matrix deposition. In neonatal mouse cardiac fibroblasts exposed to fibrotic stimuli such as arsenic trioxide, angiotensin II, or serum, PML SUMOylation and PML NB number change dynamically. Manipulating UBC9 and RNF4 expression under fibrotic stimulation strongly affects TGF β1 expression and collagen production.

Silencing UBC9 lowers overall PML SUMOylation and impairs PML NB assembly. Under these conditions, the peptidyl prolyl isomerase Pin1 is not efficiently sequestered into nuclear bodies and remains in the cytoplasm. Cytoplasmic Pin1 supports high TGF β1 expression and robust collagen synthesis under fibrotic stress. In contrast, knocking down RNF4, which normally recognizes poly-SUMOylated PML and promotes its ubiquitination and degradation, increases the number and size of PML NBs. Pin1 sequestration is enhanced, TGF β1 levels fall, and collagen accumulation is reduced. Mechanistically, this suggests that PML SUMOylation alters Pin1 localization and thereby tunes the TGF β1/collagen fibrotic program.

In mouse models of cardiac fibrosis, modulating UBC9 or RNF4 changes interstitial collagen content and alters ventricular compliance. These in vivo data support the idea that the UBC9/PML/RNF4-dependent regulation acts as a SUMO-dependent rheostat for fibrotic remodeling. PML SUMOylation and RNF4-mediated PML turnover thus sit at the center of a nuclear mechanism that translates oxidative and neurohumoral stress into long-term structural changes in the heart [55].

#### 4.2.4. SUMO-Modified Nuclear Receptors in MI/R

Myocardial ischemia–reperfusion (MI/R) injury provokes excessive cardiomyocyte apoptosis and enlarges infarct size. It also drives adverse post-infarction remodeling [56]. Several nuclear receptors and transcription factors that modulate MI/R outcomes are regulated by SUMOylation [57].

Farnesoid X receptor (FXR) is SUMOylated in cardiac tissue at baseline. FXR SUMOylation declines during ischemia and reperfusion in mouse hearts. The overexpression of a SUMOylation-defective FXR mutant worsens MI/R injury. Hearts expressing this mutant show stronger activation of the mitochondrial apoptotic pathway, impaired autophagy, and larger infarcts than hearts expressing wild type FXR. Mechanistic study indicates that, under ischemia–reperfusion stress in the heart, reduced FXR SUMOylation increases FXR transcriptional activity and increases SHP expression, which in turn drives pro-apoptotic response [58].

Peroxisome proliferator-activated receptor γ (PPARγ) is another SUMO-modified nuclear receptor with relevance to MI/R. During MI/R, the downregulation of the E3 ligase PIAS1 reduces PPARγ SUMOylation. As a result, PPARγ loses part of its ability to repress NF κB and to exert anti-apoptotic and anti-inflammatory actions, which aggravates myocardial injury [59]. These examples indicate that the SUMOylation of nuclear receptors functions as a brake on their pro-apoptotic and pro-inflammatory activities in MI/R [60]. The loss of SUMO marks under severe oxidative stress shifts the balance toward cell death and inflammation. Targeting the SUMO-dependent regulation of FXR, PPARγ, or related factors may therefore offer a complementary approach to limit reperfusion injury and adverse remodeling after myocardial infarction [57].

### 4.3. Cancer

SUMOylation regulates recurrent cancer programs across multiple functional layers. In cancer cells, the SUMO machinery is frequently engaged to stabilize signaling networks that support proliferation, stress-tolerant DNA replication, adaptation to hypoxia or nutrient limitation, epithelial plasticity, stem-like persistence, and immune evasion. At the same time, it weakens tumor-suppressive outputs such as RB- or p53-dependent cell-cycle arrest, apoptosis, and anti-tumor immune surveillance [61,62,63,64]. Consistently, SUMO modification of transcription factors, chromatin regulators, and RNA-binding proteins reprograms transcriptional output and chromatin states, supporting sustained proliferation, resistance to apoptosis, metabolic rewiring, and epithelial–mesenchymal transition (EMT) [61,62,65]. Mechanistically, this occurs because SUMOylation can alter the stability, subcellular localization, chromatin association, and co-regulator binding of transcription factors, DNA repair proteins, and metabolic regulators, thereby reshaping proliferative, DNA damage response and stress-adaptive programs [61,62,63]. SUMOylation also modulates key transcriptional and stress-response regulators, thereby reinforcing stress-adapted cell states. Accumulating clinical and transcriptomic evidence highlights the clinical relevance of the SUMO pathway, demonstrating that SUMO-related gene expression patterns are associated with tumor stratification and adverse patient outcomes, including poorer survival and higher recurrence rates, across multiple cancer types [66,67,68].

Although the precise pattern of SUMO pathway alterations and SUMO-dependent substrates varies across tumor types and molecular subgroups, a recurring theme is that increased SUMO conjugation capacity broadens the range of oxidative, genotoxic, and metabolic stress that tumor cells can tolerate and thereby favors malignant evolution. Importantly, oncogenic advantage can arise from either globally increased SUMOylation or selective shifts in deSUMOylation at specific substrates, depending on cellular context, consistent with cancer reflecting rewired SUMO homeostasis rather than a uniformly up- or down-regulated pathway. Table 1 summarizes representative SUMO-dependent modes of regulation across cancer-related transcriptional and signaling regulators.

#### 4.3.1. Cell-Cycle Control and Proliferation

SUMOylation interfaces with core cell-cycle regulators and oncogenic transcriptional programs to support G1/S and G2/M progression, attenuate stress-induced cell-cycle arrest, and weaken cell-cycle surveillance under stress. Mechanistically, RB SUMOylation in early G1 can promote CDK2 engagement and RB hyperphosphorylation, facilitating E2F release and thereby weakening RB-dependent restraint at the G1/S transition [91]. CDK6 is also directly SUMOylated in glioblastoma, where SUMO1 conjugation stabilizes CDK6 and supports cell-cycle progression [92]. Beyond individual regulators, oncogenic MYC can impose a cancer-specific dependency on SUMO E1 activity (SAE1/2), indicating that SUMO conjugation capacity becomes functionally rate-limiting for MYC-driven tumor programs [93]. SUMOylation further modulates mitotic transcriptional control through FOXM1 in a context-dependent manner, influencing mitotic progression and drug responses [63,94]. Together, these findings indicate that SUMOylation promotes continued proliferation by stabilizing cell-cycle drivers and limiting stress-induced arrest.

#### 4.3.2. DNA Damage Response and Genome Stability

Many DNA repair and replication factors are SUMO substrates, and SUMO-dependent modification can be required for timely recruitment to DNA lesions and productive repair-foci dynamics. BRCA1 is SUMOylated in response to genotoxic stress and localizes to DNA damage sites with SUMO and UBC9, linking SUMO signaling to repair assembly [95]. Under replication stress, SUMO2/3 modification of RPA70 promotes RAD51 recruitment and homologous recombination [96].

SUMO–ubiquitin coupling provides an additional control layer: RNF4, a SUMO-targeted ubiquitin ligase, recognizes poly-SUMOylated substrates at damage sites and promotes the turnover of factors such as MDC1 and RPA, enabling appropriate factor exchange during homologous recombination [30,31,37]. Together, these mechanisms support tolerance to oncogene-driven replication stress and contribute to genome stability compatible with clonal expansion while still permitting mutational diversification. Thus, the biological consequence of SUMOylation in this setting is maintenance of DNA replication and repair under genotoxic stress.

#### 4.3.3. Hypoxia Adaptation and Metabolic Reprogramming

SUMO signaling is inducible under hypoxia and can tune transcriptional programs required for survival in oxygen- and nutrient-limited tumor regions. HIF-1α is SUMOylated at lysine 391 and lysine 477, and SUMO modification has been reported to regulate HIF-1α stability and transcriptional activity [97,98]. Metabolic co-regulators are also SUMO targets: PGC-1α SUMOylation attenuates its transcriptional coactivator function, providing a mechanism to fine-tune mitochondrial and oxidative metabolic gene programs [99]. Through such nodes, SUMO-dependent regulation can support angiogenic factor production and metabolic plasticity that help sustain viability under hypoxic stress. In biological terms, this links SUMOylation to tumor survival under oxygen and nutrient limitation by influencing angiogenic signaling, mitochondrial gene expression, and metabolic plasticity.

#### 4.3.4. Invasion, Metastasis, and EMT

SUMOylation modulates EMT-associated transcription factors and signaling nodes to promote invasive phenotypes. The SUMOylation of Snail1 at a defined lysine confers transcriptional activity and is required for migratory and invasive properties in cancer cells, with downstream induction of canonical EMT genes and TGF-β pathway components [100]. SUMO-dependent stabilization of TWIST family factors has also been linked to mesenchymal programs; for example, ZNF451 promotes EMT through SUMOylation-dependent stabilization of TWIST2 [101]. In addition, SUMO pathway perturbation can influence adhesion programs, as shown by UBC9-dependent control of E-cadherin integrity in epithelial cancer models [102]. Together, these observations connect SUMO-dependent regulation to loss of epithelial adhesion, increased motility, and dissemination-associated remodeling that underlie metastatic progression.

#### 4.3.5. Cancer Stem Cells and Tumor Microenvironment

SUMOylation supports stem-like programs and microenvironmental stress adaptation that contribute to therapy resistance and relapse. In colorectal cancer models, cancer stem cell (CSC) populations show higher SUMO E1 and higher global SUMOylation than non-CSCs; genetic suppression of SUMO E1/E2 reduces CSC maintenance and tumor initiation capacity [103]. Consistent with a functional dependency, pharmacological targeting of SUMOylation reduces CSC-like self-renewal, as reflected by decreased tumorsphere formation and reduced tumor-initiating capacity in CSC-enriched models [104]. In the tumor microenvironment, activated SUMOylation can also shape anti-tumor immunity: increased SUMOylation has been shown to restrict MHC class I antigen presentation and thereby promote immune evasion [105]. Together, these findings position SUMO-dependent regulation as a determinant of stem-like persistence, reduced immune recognition, and relapse-prone tumor states under chronic stress.

### 4.4. Diabetes and Metabolic Disease

Chronic overnutrition, obesity, and diabetes expose multiple tissues to sustained metabolic stress, characterized by fluctuations in glucose and lipid availability. SUMOylation regulates proteins involved in insulin secretion, insulin signaling, lipid metabolism, inflammatory signaling, and mitochondrial function [106,107,108]. Depending on the tissue and substrate, these changes can either preserve metabolic homeostasis or contribute to β-cell dysfunction, insulin resistance, steatosis, and diabetic complications.

#### 4.4.1. SUMO-Modified MafA and β-Cell Fate

In pancreatic β-cells, SUMOylation influences the transcriptional network that maintains insulin production and β-cell identity. MafA SUMOylation at defined lysines reduces insulin promoter transactivation and shifts its target gene profile, especially under glucolipotoxic or oxidative stress. These changes parallel a transition from a mature β-cell program to one associated with dysfunction and dedifferentiation, suggesting that MafA SUMOylation acts as a switch between adaptive and maladaptive β-cell states.

More broadly, the SUMOylation of β-cell transcription factors, ER chaperones, and UPR components can bias stress responses toward apoptosis, while loss of SUMO conjugation increases mitochondrial oxidative stress and impairs respiration. β-cell SUMOylation therefore functions as a rheostat that helps determine whether glucolipotoxic stress leads to reversible adaptation or irreversible loss of β-cell mass and function [108,109].

#### 4.4.2. PPARγ, SREBP1, and NEMO in Insulin Resistance and Inflammation

In adipose tissue, PPARγ SUMOylation modifies co-regulator interactions and target gene selectivity, and the manipulation of SUMO sites in vivo suggests that specific SUMO marks can separate insulin sensitizing actions from adipogenic and weight-promoting effects. In hepatocytes, the SUMOylation of SREBP1 restrains lipogenesis at baseline, whereas reduced SUMOylation in hyperglycemic or lipid-rich states enhances lipogenic gene expression and promotes fatty liver and systemic insulin resistance.

Adipose inflammation adds a SUMO layer via NF κB control. NEMO (IKKγ) SUMOylation and its removal by SENP1 limit NF κB-driven cytokine production, whereas impaired deSUMOylation sustains inflammatory signaling, increases β-cell toxic cytokines, and produces diabetes-like phenotypes in experimental models. These findings indicate that the SUMO-sensitive regulation of PPARγ and SREBP1 links nutrient excess to insulin resistance, while NEMO-dependent inflammatory signaling provides an additional route to islet stress [110,111].

#### 4.4.3. Neuronal and Vascular SUMOylation in Diabetic Complications

In sensory neurons, reduced SUMO conjugation, for example, after conditional loss of the SUMO E2, impairs mitochondrial enzyme function, increases ROS, and accelerates peripheral nerve degeneration in type 2 diabetes models. SUMOylation of mitochondrial malate dehydrogenase 2 supports respiratory chain integrity and limits oxidative damage, highlighting a SUMO–ROS–mitochondria axis that shapes susceptibility to diabetic neuropathy.

In endothelial and renal cells, altered deSUMOylase activity modulates oxidative stress handling, inflammatory activation, and survival under hyperglycemia, contributing to glomerular injury and macrovascular complications. These observations extend the role of SUMOylation in metabolic disease from glucose homeostasis to diabetic end organ damage [109].

#### 4.4.4. SUMO–NF κB Crosstalk in Inflammatory Metabolic Stress

NF-κB signaling is central to inflammatory and metabolic stress responses in obesity and diabetes, and several pathway components are SUMO substrates. Depending on the residue, SUMO paralog, and context, the SUMOylation of IκBα, NF-κB subunits, or adaptor proteins can dampen or enhance NF-κB activity [80]. In adipocytes, the SUMOylation state of NEMO and its deconjugation by SENP1 determine the magnitude and duration of cytokine production in response to nutrient excess and innate immune stimuli.

High glucose and oxidative stress further reshape this SUMO–NF-κB crosstalk in renal and vascular cells, shifting transcription toward chronic inflammation and contributing to diabetic nephropathy and vascular dysfunction. Both excessive global SUMOylation and impaired deSUMOylation can prolong NF-κB activation, turning a transient stress response into persistent low-grade inflammation. Thus, SUMOylation tunes inflammatory signaling thresholds in a cell type-specific manner.

Beyond classical insulin-target tissues, SUMO control also extends to pancreatic, hepatic, adipose, immune, and epithelial compartments. Collectively, these SUMO-dependent effects on metabolic, inflammatory, and mitochondrial regulators contribute to tissue dysfunction and organ-specific complications (Table 2).

### 4.5. Reproduction and Fertility

Oxidative stress is repeatedly implicated in reproductive disorders in both men and women [112,113,114,115]. Protein regulation in germ cells often relies on rapid post-translational control, and SUMOylation is increasingly viewed as a plausible interface between redox fluctuations and fertility-relevant cell programs [116,117,118]. Within reproductive biology, the clearest links currently connect ROS–SUMO crosstalk to gametogenesis and germ-cell quality control, meiotic chromosome regulation, developmental competence, and the hormone-responsive pathways that support early pregnancy.

**Table 2:** SUMO-regulated regulators in metabolic and inflammatory diseases.

Tissue	Gene	Protein Pathway	SUMO-Dependent Effect	References
β-cell/islet	*MafA*	β-cell transcription factor	• Insulin promoter transactivation ↓ • β-cell gene program suppression • Dysfunction/dedifferentiation under ROS stress	[108]
	*NRF2*	Antioxidant transcription factor	• Stability ↑; antioxidant program support • ROS protection vs. secretion balance	[119]
	*PDIA3*	ER chaperone (PDI)	• Proinsulin misfolding ↑; ER stress ↑ • β-cell apoptosis ↑; insulin secretion ↓	[120]
	*Ubc9*	E2 conjugating enzyme	• Loss → mitochondrial dysfunction; ROS ↑ • Spontaneous diabetes	[119]
Liver	*HNF4α*	Hepatocyte nuclear factor	• Hepatocyte proliferation and regeneration restraint • Energy metabolism reprogramming	[121]
	*FXR*	Nuclear receptor	• Inflammatory gene modulation • Metabolic–inflammatory crosstalk tuning	[122,123]
Liver/mitochondria	*Sirt3*	Mitochondrial deacetylase	• SENP1-dependent deSUMOylation • Mitochondrial homeostasis support • Oxidative damage ↓ in hepatic I/R	[124]
Adipose tissue	*PPARγ (K107)*	Nuclear receptor	• SUMOylation physiologic • Blockade → insulin sensitivity ↑ without weight gain	[110]
	*SREBP1c*	Lipogenic transcription factor	• PIASy-mediated SUMOylation • Hepatic lipogenesis restraint	[111]
	*C/EBPβ*	Adipogenic/browning regulators	• SENP2-mediated deSUMOylation • Adipose browning suppression • SUMOylated form supports browning/beiging	[125]
Adipose/immune	*NEMO (IKKγ)*	NF-κB adaptor	• Adipocyte SENP1 loss → NEMO SUMOylation ↑ • NF-κB activation ↑; cytokines ↑ • Islet toxicity and diabetes-like phenotypes	[83]
Immune/ T-cell	*c-Maf*	Th/Tfh transcription factor	• SUMOylation → Il21 transactivation restraint • SUMO-defective c-Maf → IL-21 ↑ • Autoimmune diabetes acceleration in NOD	[126]
Intestine	*Ubc9, SUMO2/3*	SUMO machinery in intestinal epithelium	• Epithelial architecture and barrier maintenance • I/R injury responses modulation	[127,128]
Innate immunity/inflammasome	*NLRP3, MAPL, SENP6/7*	Inflammasome, E3, deSUMOylase	• MAPL-mediated SUMOylation suppresses inflammasome • SENP6/7-mediated deSUMOylation upon stimulation promotes activation	[129]

SUM ↓ indicates decreased activity/expression. ↑ indicates increased activity/expression; ↓ indicates decreased activity/expression; → indicates an ensuing outcome.

#### 4.5.1. Gametogenesis and Germ-Cell Quality Control

A prospective clinical study in couples with unexplained infertility undergoing intrauterine insemination (IUI) reported a relationship between endogenous H_2_O_2_ and abnormal accumulation of SUMO1 on defective spermatozoa [116]. Key observations were twofold: intra-sperm H_2_O_2_ levels tracked with the enrichment of SUMO1-positive abnormal sperm, and incomplete removal of defective cells during density gradient centrifugation (DGC)—leaving them in the motile fraction—was associated with poorer live-birth outcomes. These results position SUMO1 staining as a candidate readout that may track oxidative stress-linked sperm defects in a clinically relevant condition [116].

A critical point is that ROS are not uniformly harmful. A mechanistic study framework in sperm biology supports a “physiological vs. excessive” ROS continuum: low, controlled ROS participate in capacitation signaling, whereas excess ROS promotes lipid peroxidation, DNA fragmentation, and protein oxidation [112,113,117]. Consistent with this view, human sperm studies reported that SUMO1 is enriched in morphologically abnormal and poorly motile spermatozoa, correlates with sperm DNA fragmentation, and increases after oxidative or freezing–thaw stress; DRP1, RanGAP1, and Topoisomerase IIα were identified as SUMO1-associated targets, linking aberrant sperm SUMOylation to mitochondrial dysfunction, defective motility, and DNA damage rather than to a uniformly physiological modification state [116,117,130].

In the female germline, SUMOylation also acts directly on gametogenic transcriptional programs. SUMO2/3 modification of the oocyte transcription factors SOHLH1 and NOBOX alters SOHLH1 localization and promoter-specific NOBOX activity [118], and oocyte-specific loss of Ube2i in primordial follicles causes female infertility with defects in folliculogenesis, ovulation, and meiosis, accompanied by altered expression of NOBOX and its target genes [131].

#### 4.5.2. Oocyte Maturation and Meiotic Chromosome Control

In the ovary, a SUMO-focused proteomic study mapped SUMO-2/3 conjugates in lean vs. obese mice exposed to the ovotoxicant 7,12-dimethylbenz[a]anthracene (DMBA) [132]. Using LC–MS/MS, the authors identified 114 SUMO-2/3-modified ovarian targets and showed that obesity and DMBA exposure reshape SUMOylation patterns in partially distinct ways [132]. This supports the idea that the ovarian SUMOylome can behave as a dynamic stress-responsive layer, sensitive to both metabolic state and genotoxic exposure.

SUMOylation also appears to act directly on the meiotic apparatus. A mechanistic study in mouse oocytes demonstrated that the SUMO-specific regulation of Polo-like kinase 1 (PLK1) contributes to spindle integrity and kinetochore–microtubule attachment control [133]. Complementing this, another mechanistic study reported that Aurora-B kinase (AURKB) is modified by SUMO-2/3 in mouse oocytes and that SUMOylation-site mutation disrupts spindle formation and chromosome alignment [134]. This mechanistic layer extends beyond individual kinases. In oocyte-specific Ube2i-deficient mice, loss of global SUMOylation during late folliculogenesis causes female sterility, failure of transcriptional silencing and chromatin condensation in fully grown oocytes, reduced repressive histone marks, spindle defects, chromosome misalignment, and metaphase-I arrest [135]. Together, these studies place SUMOylation on both key meiotic kinases and the broader SUMO machinery as a plausible regulatory layer for structural fidelity during oocyte maturation.

#### 4.5.3. Embryonic Development and Developmental Competence

Oocyte competence strongly influences in vitro fertilization (IVF) success and broader assisted reproductive technology (ART) outcomes. Recent narrative reviews synthesize evidence that excessive oxidative stress impairs mitochondrial function, destabilizes meiotic spindles, increases DNA damage, and promotes apoptosis in follicular support cells, features repeatedly linked to reduced oocyte quality and poorer IVF performance [114,115]. In this redox-sensitive environment, stress-dependent SUMO remodeling of spindle/chromatin regulators provides a mechanistically grounded route by which metabolic or toxicant-associated oxidative burden could translate into durable meiotic errors and compromised early embryogenesis.

This developmental link is supported more directly by mouse embryo studies showing that SUMO2 knockdown reduces blastocyst formation, decreases CDX2-positive cells, ectopically maintains OCT4 and NANOG expression, and lowers global H3K27me3, indicating that SUMO2 contributes to lineage allocation and epigenetic stability during preimplantation development [136].

#### 4.5.4. Early Pregnancy and Hormonal Regulation

Direct mechanistic evidence in early pregnancy is strongest for progesterone-responsive endometrial stromal cells. During the decidualization of human endometrial stromal cells, cAMP signaling attenuates ligand-dependent progesterone receptor (PR) SUMOylation, global SUMO conjugation is remodeled, and PIAS1 acts as an E3 ligase for PR; PIAS1 silencing enhances PR-dependent transcription and induces prolactin expression in progestin-treated cells [137].

Under oxidative conditions, JNK signaling promotes hyperSUMOylation and suppresses progesterone responsiveness in undifferentiated stromal cells, whereas decidual cells attenuate this response through MKP1-dependent JNK silencing; PIAS1 further couples ROS-dependent JNK activation to oxidative cell death [138,139].

Recent human data in recurrent implantation failure extend this axis by showing that EHD1 binds PRB, promotes PRB SUMOylation and ubiquitination, lowers PRB protein abundance, and weakens decidual markers, whereas SENP1 supplementation restores PRB transcriptional activity [140].

By contrast, direct evidence linking SUMOylation to estrogen signaling in the uterus remains more limited. In a mouse repair model, stromal SENP1 deletion increased ERα SUMOylation and ERα-dependent proliferative signaling, suggesting that deSUMOylation can tune uterine estrogen responsiveness, although this evidence is more directly linked to endometrial regeneration than to implantation itself [141].

## 5. Pharmacological Modulation of SUMOylation: Translational and Clinical Perspectives

Pharmacological modulation of the SUMO pathway has been studied mainly at three steps: SUMO activation by the SUMO-activating enzyme, UBC9-mediated SUMO conjugation, and SENP-mediated deSUMOylation. Early studies showed that each step can be inhibited chemically. Ginkgolic acid and anacardic acid inhibit formation of the E1–SUMO thioester [142]. Spectomycin B1 binds UBC9 and blocks formation of the E2–SUMO intermediate [143]. 2-D08 inhibits transfer of SUMO from UBC9 to substrate proteins [144]. These compounds were important for showing that the SUMO pathway is druggable, but limited potency, selectivity, and pharmacokinetic properties have prevented clinical development [142,143,144]. By contrast, subasumstat (TAK-981) is a clinical-stage SUMO E1 inhibitor. It forms a covalent SUMO–drug adduct in the catalytic site of the SUMO-activating enzyme and thereby blocks downstream SUMO conjugation [145]. Pharmacodynamic studies in patients have shown on-target inhibition of the SUMO pathway [146,147].

This strategy has been developed most actively in cancer. In preclinical models, SUMO E1 inhibition suppresses tumor growth by reducing proliferation and survival of malignant cells and by inducing type I interferon signaling [145,148]. Subasumstat also increases macrophage phagocytosis and NK-cell cytotoxicity and enhances the activity of rituximab in lymphoma models [149]. These findings are relevant to cancer immunotherapy because increased SUMOylation in tumor cells has been linked to reduced MHC class I antigen processing and presentation and to impaired CD8^+^ T-cell-mediated immunosurveillance [105,150]. In this setting, pharmacological SUMO inhibition increases tumor-cell susceptibility to immune-mediated killing and supports combination with antibody-based or other immune-directed therapies [105,149]. In early-phase clinical studies, subasumstat, either as monotherapy or in combination with rituximab, showed pharmacodynamic target engagement and preliminary anti-tumor activity in advanced solid tumors, hematologic malignancies, and relapsed/refractory non-Hodgkin lymphoma [146,147].

SENP inhibition remains at an earlier stage of development. The rationale is not global suppression of the SUMO pathway, but inhibition of deSUMOylation of proteins that promote tumor growth, metabolic reprogramming, or drug resistance. Momordin Ic is the best-characterized SENP1 inhibitor and shows antiproliferative activity in prostate cancer models [151]. In gastric cancer, SENP1 stabilizes ENO1 through deSUMOylation, promotes glycolysis, and contributes to cisplatin resistance; Momordin Ic increases ENO1 SUMOylation and enhances cisplatin sensitivity in vitro and in xenograft models [152]. SENP1-dependent drug resistance phenotypes have also been described in platinum-resistant ovarian cancer [153]. However, SENP modulators remain preclinical, and further development will require improved isoform selectivity, pharmacokinetic properties, and biomarkers of target engagement [151,152,153].

Available data indicate that the therapeutic effect of SUMO modulation depends on the protein and disease process being targeted. In cancer, current evidence supports SUMO E1 inhibition most strongly, particularly in tumors in which SUMOylation contributes to survival under replicative, proteotoxic, or therapy-induced stress and in settings in which antigen presentation or interferon signaling is impaired [105,148,149]. Genomic and transcriptomic studies further support the translational relevance of this pathway, as alterations in SUMO pathway regulators have been linked to tumor stratification and prognosis across multiple cancer types [66]. Such changes may help identify tumor subsets with increased dependence on SUMO signaling and potential vulnerability to SUMO-targeted therapy [154]. In contrast, other diseases may require the enhancement rather than inhibition of SUMOylation. Arsenic trioxide exploits SUMO-dependent ubiquitin-mediated degradation of PML–RARα in acute promyelocytic leukemia [155,156], whereas N106 increases SERCA2a SUMOylation and improves cardiac function in heart failure models [157]. These examples indicate that pharmacological modulation of SUMOylation is unlikely to rely on a single strategy across all diseases. Further clinical development will require disease-specific selection of SUMO-targeted agents and biomarkers that report pathway inhibition or substrate modification in patients.

## 6. Future Perspectives and Conclusions

Beyond malignant disease, more selective tuning of SUMO signaling is under investigation in ischemic, metabolic, inflammatory, and reproductive disorders. In renal ischemia–reperfusion and diabetic kidney models, the modulation of deSUMOylase activity alleviates oxidative stress, mitochondrial dysfunction, and tubular or podocyte apoptosis, indicating that the deSUMOylation of specific substrates can be cytoprotective in certain renal injuries. In type 2 diabetic peripheral neuropathy, the preservation of SUMOylation on mitochondrial enzymes in sensory neurons maintains respiratory chain function and restrains ROS accumulation, whereas the loss of neuronal SUMOylation exacerbates oxidative damage and nerve fiber loss.

In the reproductive system, clinical and experimental studies link ROS–SUMO crosstalk to sperm quality, ovarian stress responses, and oocyte competence. SUMO1-positive sperm subpopulations associate with oxidative stress-related sperm defects and impaired live-birth rates, ovarian SUMO-2/3 conjugates are reconfigured by obesity and ovotoxicants, and the SUMOylation of meiotic kinases such as PLK1 and AURKB supports spindle integrity and chromosome alignment during oocyte maturation. Recent studies further indicate that SUMOylation contributes to preimplantation developmental competence and progesterone-responsive decidualization, extending the relevance of ROS–SUMO crosstalk to early pregnancy. Across liver, adipose tissue, pancreatic β-cells, and reproductive tissues, genome- and proteome-wide analyses underscore that SUMO-dependent redox adaptation is highly tissue-specific, arguing for cell type-targeted modulation of SUMO rather than global activation or inhibition.

Taken together, cardiovascular, cancer, metabolic, neurological, and reproductive models depict ROS–SUMO crosstalk as a disease-relevant mechanism that can either preserve tissue integrity or promote pathology, depending on the affected substrates, SUMO paralogs, and the tissue and cell type. SUMOylation of SERCA2a, UBC9-dependent control of proteostasis, and the PML/RNF4 axis shape the balance between adaptive remodeling and decompensation in the heart, whereas in tumors, the same enzymatic machinery is repurposed to stabilize oncogenic signaling and buffer genotoxic and metabolic stress. In metabolic and reproductive organs, the SUMO-dependent modulation of transcription factors, kinases, meiotic regulators, and organelle function supports adaptation but, when mis-tuned, promotes chronic dysfunction, infertility, and age-related decline. These observations argue against viewing SUMOylation as uniformly protective or pathogenic and instead emphasize tissue-specific responses, substrate selectivity, and the level and duration of oxidative stress.

In conclusion, ROS–SUMO crosstalk is a disease-relevant mechanism that shapes how cells and tissues respond to oxidative and metabolic stress across neurodegenerative, cardiovascular, malignant, metabolic, and reproductive disorders. Future studies should define substrate-specific and paralog-specific SUMO responses in vivo, particularly in reproductive processes such as oocyte maturation, preimplantation development, decidualization, and early pregnancy, and should establish biomarkers that report pathway inhibition or substrate modification in patients. Defining when and where SUMOylation acts as a safeguard versus a facilitator of pathology, and learning to recalibrate this pathway, attenuating SUMO-dependent stress support in tumors while preserving or enhancing its beneficial roles in vulnerable organs such as the heart, kidney, nervous system, and reproductive tissues will be essential to translate rapidly expanding basic knowledge of ROS–SUMO biology into safe and effective interventions for human disease.

## Figures and Tables

**Table 1:** SUMO pathway regulators across cancer types.

Cancer Type/Tissue	SUMO-Regulated Regulators	SUMO-Dependent Effects on Function	References
Breast cancer	ESR1, PGR, ESR2, TFAP2C, MYB, MTA1, SP1	• ESR1/ESR2: stability ↑, chromatin binding modulation • PGR/MTA1: HDAC2 recruitment, target gene repression • SP1: activity and stability shifted by SUMO1 vs. SUMO2	[69,70,71,72]
Prostate cancer	AR, FOXM1, EGR1, SP1	• AR: co-regulator switching, target program activation/repression • FOXM1: negative regulatory domain masked, activity ↑ • SP1: transcriptional output and stability modulation	[72,73,74,75,76]
Hematologic malignancies (leukemia, lymphoma)	GATA2, CEBPA/B/E, MYB, IRF1, IκBα	• GATA2, CEBP family: reinforced transcriptional repression • IRF1: tumor-suppressive activity enhanced • IκBα: stronger NF-κB inhibition, degradation resistance	[77,78,79,80]
TGF-β/EMT-linked cancers (various)	SMAD4, SMAD3, CTBP	• SMAD4: stability ↑, DAXX interaction ↑, TGF-β signaling ↑ • SMAD3: DNA-binding and nuclear–cytoplasmic shuttling control • CTBP: nuclear localization and repressor complex assembly	[81,82,83]
Solid tumors (lung, colon and others)	HIF1A, HSF1/HSF2, SRF, PLAG1, ELK1, DDX5	• HSF2: DNA-binding ↑, stress-induced gene expression ↑ • SRF/ELK1/PLAG1: bias toward repression of subsets of targets • DDX5: HDAC1 interaction ↑, locus-specific repression	[84,85,86,87,88]
various cancers	EGR1, SP3, SP1	• SP3: non-SUMOylated when DNA-bound, context-specific repression • SP1: SUMO1 vs. SUMO2 with opposing effects on stability/activity	[72,89,90]

↑ indicates increased activity/expression.

## Data Availability

No new data were created or analyzed in this study. Data sharing is not applicable to this article.

## Abbreviations

The following abbreviations are used in this manuscript:

PTMs	post-translational modifications
ROS	reactive oxygen species
SUMO	small ubiquitin-like modifier
OGD	oxygen–glucose deprivation
DDR	DNA damage response
DSBs	double-strand breaks
HR	homologous recombination
E2F1	E2F transcription factor 1
MDC1	mediator of DNA damage checkpoint 1
RPA	replication protein A
RNF4	RING finger protein 4
PML	promyelocytic leukemia protein
PML-NBs	PML nuclear bodies
TDP-43	TAR DNA-binding protein 43
JNK	c-Jun N-terminal kinase
PIAS1	protein inhibitor of activated STAT 1
PIAS4	protein inhibitor of activated STAT 4
SERCA2a	sarcoplasmic reticulum Ca^2+^ ATPase
TAC	transverse aortic constriction
DRM	desmin related cardiomyopathy
CryABR120G	mutant αB crystallin
MI/R	myocardial ischemia–reperfusion
FXR	farnesoid X receptor
PPARγ	peroxisome proliferator-activated receptor γ
IUI	intrauterine insemination
DGC	density gradient centrifugation
DMBA	7,12-dimethylbenz[a]anthracene
LC–MS/MS	liquid chromatography–tandem mass spectrometry
IVF	in vitro fertilization
ART	assisted reproductive technology
CSC	cancer stem cell
